# The sociopolitical dimension of attachment: differences in attachment anxiety and attachment avoidance between East and West Germans

**DOI:** 10.1186/s40359-026-05855-x

**Published:** 2026-10-22

**Authors:** Christoph Kasinger, Katja Müller, Marie-Theresa Kaufmann, Bernhard Strauss, Manfred Beutel, Elmar Brähler

**Affiliations:** 1 Department of Psychosomatic Medicine and Psychotherapy, University Medical Center, Johannes Gutenberg University Mainz, Mainz, Germany; 2 Department for Child and Adolescent Psychiatry/Psychotherapy, University of Ulm, Ulm, Germany; 3 Jena University Hospital, Institute of Psychosocial Medicine and Psychotherapy, Jena, Germany; 4 Clinic and Polyclinic for Psychosomatic Medicine and Psychotherapy, Rostock University Medical Center, Rostock, Germany

**Keywords:** Attachment anxiety, Attachment avoidance, East-West Germany – ECR-12, Romantic relationships

## Abstract

**Background:**

Attachment is a universal, cross-cultural basic need, which, however, is defined, satisfied and shaped differently in each socio-cultural context. Germany with its history of two divided states and their different sociopolitical environments offers an unique opportunity to study their impact on attachment behavior.

**Methods:**

Attachment behavior was assessed in a representative sample (*N* = 2,328) using the dimensions of attachment anxiety and attachment avoidance in couple relationships of the Experiences in Close Relationships questionnaire (ECR-RD 12). Differences between East and West Germans were examined in a univariate analysis of variance, differentiated by age cohorts and sex. Socioeconomic variables were included in the model as control variables.

**Results:**

Significant East-West differences were found for attachment anxiety (*p* = .01), but not for attachment avoidance. The analysis differentiated by age cohorts revealed on average a lower level of attachment anxiety for East Germans born between 1921 and 1989 compared to West Germans. These differences were not found for the post-reunification generation (born after 1989), although this comparison included only 28 East German participants and should be interpreted with caution. Gender-related effects were only found in relation with age cohorts.

**Conclusion:**

Adults who grew up in the former German Democratic Republic (GDR) report lower attachment anxiety than their West German counterparts. Although effect sizes were small, the findings suggest that the sociopolitical environment is a relevant factor for attachment behavior in close relationships, particularly attachment anxiety.

## Introduction

Attachment is defined as “an emotional bond that develops between an infant and their primary caregivers” [18]. It primarily characterizes emotionally significant relationships throughout an individual’s life, such as those with parents, siblings, grandparents, close friends, and romantic or marital partners [13, 28]. Bowlby postulated that attachment is an evolutionary, universal, and distinct fundamental human need for contact and emotional security. Children are considered to have an innate capacity to form attachments with close caregivers. In addition to providing care and protection, this capacity serves the primary function of conveying a sense of safety and trust in emotionally stressful situations [6]. A substantial body of research has demonstrated that the quality of early attachment experiences significantly influences a child’s socio-emotional development [14], and that adverse attachment experiences constitute a relevant risk factor for the development of mental disorders [29]. With regard to the stability of attachment behavior, it has been shown that attachment patterns are relatively stable in adulthood (e.g., over the course of romantic partnerships; [17]). However, attachment relationships do not develop in isolation from their surrounding environment. Bronfenbrenner’s bioecological model [9], though not developed specifically for attachment, describes five interrelated systems that can be applied to an individual’s attachment development. Proximal processes — the child’s direct interactions within the family and immediate social environment (microsystem), and the links between these settings (mesosystem) — have long been the focus of attachment research. Considerably less attention has been paid to the three more distal layers, which are of primary interest here. The exosystem comprises contexts that affect the child indirectly through their influence on caregivers, most notably parental employment and childcare availability. The macrosystem refers to a society’s shared normative and value orientations, including child-rearing goals and gender-role models. The chronosystem captures the timing of development relative to historical transitions, such that a nominally identical environment can carry different developmental meaning across birth periods. East and West Germany differed systematically in exactly these three distal dimensions, making the divided country a natural setting in which to study their influence on attachment.

East and West Germany belong to the same cultural sphere, sharing common cultural and historical roots as well as a common language. However, the division of Germany in 1949 gave rise to two systems with fundamentally different economic, political, and social frameworks. While the Federal Republic of Germany (FRG) developed as a Western-style democracy, the former German Democratic Republic (GDR) evolved into a socialist state modeled after the Soviet Union, governed and monitored by an authoritarian and repressive regime. These divergent structural conditions also shaped attitudes, norms, and values [31]. In the GDR, collectivist values such as social connectedness, harmony, equality, as well as obedience and subordination, were emphasized. In contrast, in the FRG, the “freedom and dignity of the individual”—that is, individualism—was of central importance [21]. With regard to family life and parenting, differences emerged particularly in family formation, gender roles, and parenting goals. In the former GDR, the state actively promoted both early marriage and family formation (average age at first marriage: GDR = 22 years compared with FRG = 25 years; [22]) as well as the equal participation of men and women in the workforce. Consequently, the female employment rate was significantly higher in the GDR (82.8%) compared to the FRG (50%) [27]. In order to facilitate the reconciliation of family and work life, the GDR provided comprehensive, state-funded childcare services, which were widely utilized even for children under the age of three (approximately 80% in the GDR compared with fewer than 3% in the FRG; [27]). In contrast, traditional gender roles continued to shape family life in the former FRG for an extended period. Mothers typically took three or more years of parental leave and rarely returned to full-time employment, often opting instead for part-time work [27]. Early non-familial childcare was considered appropriate only in cases of adverse family circumstances [1].

Empirical evidence on East-West differences in attachment is limited [1, 2, 3]: infants in the two regions were equally likely to be securely attached to their non-parental caregivers, but insecure patterns differed in kind, with avoidant attachment more common in the East and disorganized attachment more common in the West. These studies drew on small, non-representative samples of children already enrolled in childcare facilities, and assessed attachment to the caregiver rather than to a parent; whether comparable regional differences exist in parental or romantic attachment, and whether they persist into adulthood, remains unknown.

Because the two German states existed for four decades and both changed substantially over that period, treating region of upbringing as a single, time-invariant exposure would obscure precisely the chronosystemic variation of interest. We therefore distinguish four birth cohorts, each of which encountered a distinct configuration of family policy, childcare provision, and prevailing parenting norms during its formative years [23]. The cohort boundaries correspond to documented institutional discontinuities rather than to arbitrary intervals: the founding of the two states in 1949, the consolidation of divergent family policy regimes together with the liberalization of West German parenting norms around 1968, and reunification in 1989/90. The post-war cohort (born 1921–1948) spent childhood before or during the establishment of the two states, under conditions of war, displacement, paternal absence, and authoritarian parenting ideals shared across both regions; regional differences in this cohort therefore reflect the pre-division period rather than the mature systems. The state-building cohort (born 1949–1967) was the first to be socialized under separate regimes: in the East, the consolidation of socialist family policy and the rapid expansion of female employment; in the West, economic reconstruction and the normative primacy of maternal care [22, 27]. The late-socialist cohort (born 1968–1989) grew up under the most divergent versions of both systems: by this period, around 80% of East German children under three attended state-funded external childcare, compared with fewer than 3% in the West, where non-familial childcare remained uncommon despite the post-1968 liberalization of parenting norms [1]. The post-reunification cohort (born 1990 or later) is included not to test GDR socialization but as an exploratory contrast cohort: institutional unification predicts convergence, whereas the documented persistence of GDR-formed preferences and intergenerational transmission from GDR-socialized parents predict attenuated but non-zero differences [4, 16, 32].

This former division offers a natural experiment that isolates these structural factors more cleanly than a present-day cross-country design could: two populations sharing a common language and culture were exposed for four decades to sharply different family and childcare policies. The present study investigates the extent to which these differences in socialization conditions have shaped later attachment behavior in romantic relationships among adults from East and West Germany.

## Method

### Study design and sample

This study is based on a cross-sectional design. Data were collected in 2013 through a nationwide survey conducted in Germany by the social research institute USUMA (Berlin). A multistage sampling procedure was used (sample points proportionate to national household distribution, random-route household selection, and a Kish grid for individual selection) to ensure a representative sample with respect to age, gender, and educational background. Inclusion criteria were a minimum age of 14 years and sufficient proficiency in the German language. Data were gathered through structured interviews.

A total of 2,508 individuals participated in the survey. Only cases with complete data for the key variables relevant to analysis (region, gender, age, income, education and the ECR scales for attachment anxiety and avoidance) were included. 180 participants were excluded due to incomplete responses or data coding errors, resulting in a final sample of 2,328 participants (female: *N* = 1,241; male: *N* = 1,087; West Germany: *N* = 1,868; East Germany: *N* = 460). Participants ranged in age from 14 to 92 years (M = 49.44; SD = 18.03). Further socio-demographic characteristics are presented in Table 1.

**Table 1:** Socio-demographic characteristics of the sample

		Total *N* = 2,328 (100%)	West *N* = 1,868 (80.2%)	East *N* = 460 (19.8%)
Sex	Male	1,087 (46.7%)	876 (46.9%)	211 (45.9%)
	Female	1,241 (53.3%)	992 (53.1%)	249 (54.1%)
Age	*M* (+/- *SD*)	49.44 (18.03)	48.26 (17.91)	54.22 (17.76)
Education	Yes	422 (18.1%)	341 (18.3%)	81 (17.6%)
(Uni. qualification)	No	1,906 (81.9%)	1,527 (81.7%)	379 (82.4%)
Age cohort	1921–1948	545 (23.4%)	392 (21.0%)	153 (33.3%)
(Year of birth)	1949–1967	807 (34.7%)	642 (34.4%)	165 (35.9%)
	1968–1989	769 (33.0%)	655 (35.1%)	114 (24.8%)
	1990–2004	207 (8.9%)	179 (9.6%)	28 (6.1%)
Household Income (€/ month)	below 2,000 €	1,182 (50.8%)	898 (48.1%)	284 (61.7%)
	above 2,000 €	1,146 (49.2%)	970 (51.9%)	176 (38.3%)

Note.* M = M*ean. *SD =* Standard Deviation

### Measures

Attachment behavior was assessed using the revised German short version of the Experiences in Close Relationships questionnaire (ECR-RD12; [8, 19]). The 12-item self-report measure captures two dimensions, Attachment Anxiety and Attachment Avoidance, with six items each. Participants rated items on a 7-point Likert scale (1 = strongly disagree, 7 = strongly agree), based on their general experiences in romantic relationships. Scale scores were calculated as sum values, ranging from 6 to 42 per scale. Higher scores indicate greater attachment anxiety or avoidance, while lower scores on both scales suggest attachment security. Cronbach’s α was 0.88 for the Attachment Anxiety scale and 0.86 for the Attachment Avoidance scale, indicating high internal consistency for both scales.

Regional classification was based on the participants’ place of residence (East or West Germany; former GDR or former FRG respectively) up to the age of 14.

Consistent with the rationale set out in the Introduction, participants were assigned to four birth cohorts: the post-war cohort (1921–1948), the state-building cohort (1949–1967), the late-socialist cohort (1968–1989), and the post-reunification cohort (1990–2004).

Sociodemographic variables included sex as assigned at birth, the equivalized household income according to OECD guidelines (household income divided by the square root of household size; OECD, 2011), and education (holding a German university entrance qualification). The household income variable was dichotomized at €2,000 per month, which closely approximates the median of the equivalized household income in the present sample (below €2,000: 50.8%; above €2,000: 49.2%).

### Statistical analysis

To investigate the influence of East-West socialization on attachment behavior, univariate analyses of variance were conducted. East-West differences were examined with regard to attachment anxiety and attachment avoidance, differentiated by four birth cohorts (1921–1948; 1949–1967; 1968–1989; 1990–2004) and by sex (male/female), and controlled for education and household income. East-West differences differentiated by birth cohort were further specified with post-hoc tests. Because Levene’s test indicated significant heterogeneity of variance across the eight East/West × cohort groups, Games-Howell tests, which are robust to both unequal sample sizes and unequal variances, were used. The significance level was set at α = 0.05. All statistical analyses were conducted using the open-source statistical software JASP (JASP Team, 2024; Version 0.19.1).

## Results

Table 1 shows relevant socio-demographic characteristics of the sample.

Table 2 presents the results of the univariate analysis of variance with the independent variables East/West, age cohorts and sex, and their association on attachment anxiety and attachment avoidance as dependent variables, controlled for income and education. There were significant East-West differences for attachment anxiety (*F*(1, 2,310) = 7.40, *p* = .01), but not for attachment avoidance. The age cohort effect was significant for both, attachment anxiety (*F(*3, 2,310) = 21.08, *p*<.01) and attachment avoidance (*F*(3, 2,310) = 4.07, *p <* .01). Within the control variables, education and income showed a significant association for both variables.

**Table 2:** Results of the univariate analysis of variance for attachment anxiety and attachment avoidance

	Sum of Squares	df	Mean Square	F	*p*	η²_part_
**Attachment anxiety**						
East/West	303.63	1	303.63	7.40	0.01*	< 0.01
Age cohort	2,594.44	3	864.81	21.08	< 0.01*	0.03
Sex	21.56	1	21.56	0.53	0.47	< 0.01
Education	368.01	1	368.01	8.97	< 0.01*	< 0.01
Household Income	1,058.36	1	1,058.36	25.80	< 0.01*	0.01
East/West * age cohort	376.01	3	125.34	3.06	0.03*	< 0.01
East/ West * sex	0.25	1	0.25	0.01	0.94	< 0.01
Age cohort * sex	380.22	3	126.74	3.09	0.03*	< 0.01
East/West * age cohort * sex	200.11	3	66.70	1.63	0.18	< 0.01
Residuals	94,749.47	2,310	41.02			
**Attachment avoidance**						
East/ West	6.77	1	6.77	0.10	0.75	< 0.01
Age cohort	834.74	3	278.25	4.07	< 0.01*	0.02
Sex	22.55	1	22.55	0.33	0.57	< 0.01
Education	338.81	1	338.81	4.95	0.03*	< 0.01
Household Income	9,051.10	1	9,051.10	132.26	< 0.01*	0.06
East/West * age cohort	433.70	3	144.57	2.11	0.10	< 0.01
East/ West * sex	177.05	1	177.05	2.59	0.11	< 0.01
Age cohort * sex	260.10	3	86.70	1.27	0.28	< 0.01
East/ West * age cohort * Sex	211.08	3	70.36	1.03	0.38	< 0.01
Residual	158,088.13	2,310	68.44			

Note. * p<.05. η²_part_: small 0.01; medium 0.06; large 0.14

**Fig. 1:**
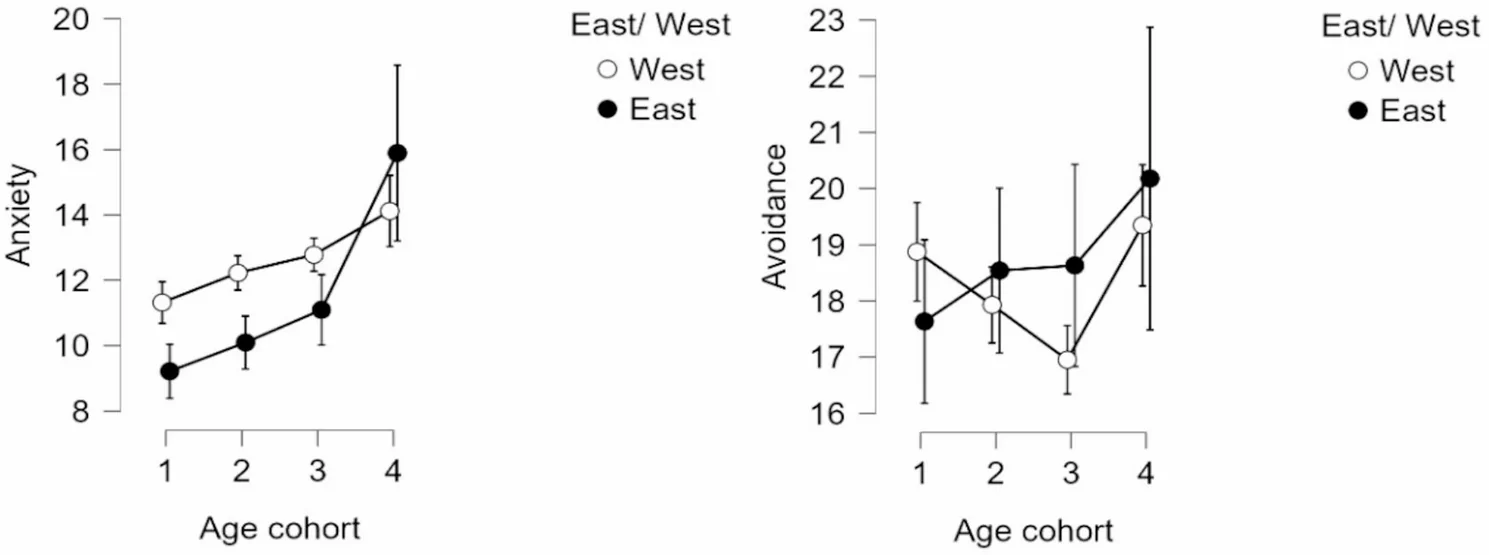
East-West differences in attachment anxiety and attachment avoidance, differentiated by age cohorts (1: born 1921–1948; 2: born 1949–1967; 3: born 1968–1989; 4: born 1990–2004)

To specify the interaction effect of location and age cohorts, a more nuanced investigation was conducted. Results of the East-West comparisons by age cohorts are presented in Table 3; Fig. 1.

**Table 3:** Results of the post-hoc tests: East-West differences in attachment anxiety and attachment avoidance, differentiated by age cohorts

			95% CI						
	Mean Difference		Lower	Upper		SE		*P*__GH_	
**Attachment anxiety**									
Age cohort 1: 1921–1948	1.89		0.33	3.46		0.51		< 0.01*	
Age cohort 2: 1949–1967	2.11		0.64	3.58		0.48		< 0.01*	
Age cohort 3: 1968–1989	1.82		0.02	3.61		0.59		0.04*	
Age cohort 4: 1990–2004	-1.19		-5.28	2.89		1.29		0.98	
**Attachment avoidance**									
Age cohort 1: 1921–1948	1.18		-1.40	3.75		0.84		0.86	
Age cohort 2: 1949–1967	-0.57		-3.05	1.91		0.81		1.00	
Age cohort 3: 1968–1989	-1.33		-4.17	1.51		0.92		0.84	
Age cohort 4: 1990–2004	-0.77		-5.16	3.62		1.38		1.00	

Note.* CI* = Confidence Intervals. *SE* = Standard Error. *p__GH_ < 0.05

As shown in Fig. 1, East-West differences in attachment anxiety and attachment avoidance varied depending on the age cohort. However, the interaction term between location and age cohorts only showed a significant effect for attachment anxiety (*F*(3, 2,310) = 3.06, *p* = .03).

In each of the three oldest cohorts, East German participants reported significantly lower attachment anxiety than West German participants (mean differences of 1.82 to 2.11 points, all p_GH < 0.05): the post-war, state-building, and late-socialist cohorts. For attachment avoidance, no significant difference between East and West Germans emerged.

Regarding gender-related effects, a significant interaction between sex and birth cohort emerged only for attachment anxiety, F(3, 2,310) = 3.09, *p* = .03, but not in relation to East-West socialization.

## Discussion

This study examined whether the distinct socialization conditions in formerly divided Germany (1949–1989) have led to differences in adult attachment behavior between individuals from East and West Germany. The findings indicate that individuals who were socialized in the former GDR exhibit significantly lower levels of attachment anxiety compared to their West German counterparts. In contrast, no significant regional difference in attachment anxiety was found for the post-reunification generation, although the small sample size of this cohort (*n* = 28 East German participants; see Limitations) limits the conclusions that can be drawn. No significant differences were found between East and West Germans with respect to attachment avoidance.

Several explanations for this pattern are considered below. A methodological caveat applies to all of them: the ECR-RD12 is a self-report measure, and low attachment anxiety scores cannot be distinguished from a learned habit of suppressing vulnerability. Such a habit could have several sources, from a broader cultural norm of emotional restraint to specific caution around self-disclosure in a surveilled society [20]. Notably, however, the East-West difference was confined to attachment anxiety and largely absent for avoidance, which a general response-style bias would not predict.

One potential explanation for the greater attachment security observed among individuals socialized in East Germany may lie in more positively perceived parenting experiences. Beutel et al., [5] found that East Germans recalled their parents as less strict, controlling, and overprotective, and described their fathers as more emotionally warm compared to West German respondents. Supporting this, Schulz et al., [25] reported significantly fewer adverse childhood experiences among individuals raised in the former GDR. Both findings suggest a more favorable parent-child relationship quality in East Germany, which is a well-established predictor of secure attachment development [7] and has been shown to correlate with secure attachment behavior in adult romantic relationships [26].

Additionally, a more egalitarian female role and less conservative partnership norms may also have fostered greater attachment security among East Germans, thereby helping to explain the differences observed in comparison with West German adults. In the former Federal Republic of Germany, traditional gender roles dominated family life: mothers assumed primary responsibility for child-rearing, which largely took place within the core family. By contrast, in the German Democratic Republic, the widespread employment of mothers embedded child-rearing from the outset within broader, tightly knit social networks. Consequently, East German children formed meaningful relationships with multiple caregivers, a pattern shown to promote later attachment security when those relationships are of adequate quality [24]. This benefit was not universal, however: the GDR also relied on weekly and seasonal childcare institutions involving extended overnight separation from parents (Wochenkrippen, Saisonkrippen), and adults who experienced this form of care show poorer attachment outcomes [11]. Huth-Bocks et al., [15] likewise demonstrated that maternal social support—such as an extensive childcare network—is positively associated with the quality of mother–child attachment. More generally, social cohesion in the GDR, reinforced both by material shortages and as a counterweight to an authoritarian regime, held considerable functional and emotional significance [10]. This strong perceived interconnectedness may have further facilitated attachment security in the GDR, though the same collectivist structures could equally be read as enforced conformity rather than freely chosen social integration.

Structural conditions of partnership formation may offer a further, non-clinical explanation. Housing scarcity in the GDR made a stable partnership a practical precondition for obtaining a separate apartment, contributing to markedly earlier marriage (see above); relationships formed under this pressure may have settled into more predictable, lower-conflict patterns by the time they were reported on retrospectively.

Apart from early childhood attachment experiences, relational experiences during adolescence and early adulthood play a formative role in shaping later romantic attachment behavior [12]. Here, the specific socialization context in the former GDR—characterized by shared educational trajectories (e.g., joint schooling until the 10th grade) and a high degree of institutionalized leisure activities—likely fostered close, trusting peer relationships among adolescents, which may have contributed to greater interpersonal security and attachment stability in adulthood. However, much of this shared leisure activity took place within state-organized youth structures (e.g., the Pioneer organization, Free German Youth) that emphasized collective conformity, so the same closeness could equally reflect socially enforced cohesion rather than a genuine developmental advantage.

In summary, the observed differences in adult attachment behavior between East and West German participants suggest a potential influence of region-specific socialization experiences. However, with regard to the small effect sizes the influence of these experiences may be limited. Additional factors likely contribute to the relationship between attachment patterns in adulthood and early socio-relational experiences shaped by the former East–West divide.

### Limitations and outlook

It is questionable whether measuring adult attachment solely along the dimensions of attachment anxiety and avoidance within romantic relationships is sufficient to validly infer more general internalized attachment patterns. Second, there remains a lack of consensus on the appropriate operationalization and assessment of adult attachment. While interviews and self-report questionnaires each have methodological strengths and limitations, research has shown that these approaches may tap into different levels of attachment-related experiences, potentially leading to divergent outcomes [30].

Furthermore, internal migration, particularly from East to West Germany following reunification, was not accounted for in the present study. Regional classification was based on place of residence up to age 14, but not on current residence. It is therefore conceivable that adult attachment behavior may also have been shaped by sociocultural influences encountered after migration, particularly within West German contexts. This is particularly relevant for the post-reunification cohort: participants born in the West to parents who had migrated from the East after 1989 are classified as West German, even though intergenerational transmission of attachment representations from GDR-socialized parents could shape their attachment behavior in the same direction as the older East German cohorts; to the extent this occurs, it would dilute rather than inflate the observed East-West differences. Future studies should record full residential and parental migration histories to test this directly.

An additional limitation is that household income was dichotomized at €2,000 per month (approximating the sample median), which may have reduced statistical precision and left residual socioeconomic differences between regions incompletely controlled.

Additionally, East and West German participants differed slightly in age within some cohorts. A sensitivity analysis adding continuous age to the reported model confirmed a small residual age effect within cohorts, but the East-West difference in attachment anxiety remained significant, indicating it is not an artifact of residual age confounding. Finally, the small number of East German participants born after 1990 (post-reunification cohort; *n* = 28) also represents a limitation of this study. This sample size provides limited statistical power to detect an effect of the magnitude observed in the older cohorts, so the absence of a significant East-West difference in this cohort should be interpreted with caution rather than as evidence of convergence. Relatedly, the overall regional split (80% West, 20% East) reflects the population distribution but further limits statistical power for the East German subsample specifically, and asymmetric group sizes can make the assumptions underlying between-group tests harder to satisfy.

To gain a deeper understanding of the complex interplay between individual and sociocultural factors, further research is warranted. Building on the findings of the present study, future research could focus on how differing norms and values related to partnership and gender roles influence attachment behavior. Such studies might also draw connections to experiences in other post-socialist societies. Moreover, subsequent research could conceptualize adult attachment more broadly, extending beyond romantic partnerships to include other domains of attachment behavior, such as relationships with one’s own children, attachment within professional settings, or the role of social networks.

## Data Availability

The dataset analysed in the current study is not publicly available due to data protection and contractual restrictions. Data may be made available from the corresponding author upon reasonable request and subject to the approval of the data owner.
